# Exploration of Pea Protein Isolate–Sodium Alginate Complexes as a Novel Strategy to Substitute Sugar in Plant Cream: Synergistic Interactions Between the Two at the Interface

**DOI:** 10.3390/foods14060991

**Published:** 2025-03-14

**Authors:** Jingru Sun, Xiyuan Yang, Jingjing Diao, Yichang Wang, Changyuan Wang

**Affiliations:** 1 College of Food, Heilongjiang Bayi Agricultural University, Xinfeng Road 5, Daqing 163319, China; s15545723965@163.com (J.S.); 13555517359@163.com (X.Y.); diaojing62@163.com (J.D.); 2 National Coarse Cereals Engineering Research Center, Daqing 163319, China; 3 College of Food Science, Northeast Agricultural University, Harbin 150030, China; 13351999556@163.com

**Keywords:** pea protein isolate, sodium alginate, whipped cream, interface characteristics, synergistic interactions

## Abstract

This study aims to explore the feasibility of using pea protein isolate (PPI)/sodium alginate (SA) complex as a sugar substitute to develop low sugar plant fat cream. Firstly, this study analyzed the influence of SA on the structure and physicochemical properties of PPI and evaluated the types of interaction forces between PPI and SA. The addition of SA effectively induces the unfolding and structural rearrangement of PPI, causing structural changes and subunit dissociation of PPI, resulting in the exposure of internal-SH groups. In addition, the addition of SA increased the content of β-folding in PPI, making the structure of PPI more flexible and reducing interfacial tension. The ITC results indicate that the binding between PPI and SA exhibits characteristics of rapid binding and slow dissociation, which is spontaneous and accompanied by heat release. Next, the effect of PPI/SA ratio on the whipping performance and quality of low sugar plant fat creams was studied by using PPI/SA complex instead of 20% sugar in the cream. When using a PPI/SA complex with a mass ratio of 1:0.3 instead of sugar, the stirring performance, texture, and stability of plant fat cream reach their optimum. Finally, the relevant analysis results indicate that the flexibility and interface characteristics of PPI are key factors affecting the quality of cream. This study can provide theoretical support for finding suitable sugar substitute products and developing low sugar plant fat cream.

## 1. Introduction

Plant fat cream is a product with complex oil–water emulsion structure formed by mixing, homogenizing, cooling, and stirring plant fat and sugar as raw materials, adding protein, emulsifier, hydrocolloid, water, and other components [1]. Plant fat cream has the characteristics of low cost, good stability, and easy processing. It is very well known in food processing fields such as baking and catering. It is widely used in the production of various processed foods, such as ice cream, cakes, coffee, etc. Traditional fat cream contains 25–30 wt% of sugar compounds. Its high sugar content and high glycemic index will increase the risk of many health problems, such as obesity or type Ⅱ diabetes [2]. Simply reducing the sugar compounds in fresh cream is the most direct and simple way to reduce health risks. However, simply reducing the amount of sugar will have adverse effects on the quality and acceptability of its fresh cream due to the decrease in soluble solids content and viscosity [3]. Cream will exhibit extremely low hardness and stability. At this point, many formulas have been developed to replace sugar in the production of healthier and higher quality low sugar plant cream [4].

Protein–polysaccharide complexes can serve as excellent carbohydrates substitutes, as their sweet taste characteristics and low impact on blood sugar levels can simultaneously meet consumer taste expectations and better control blood sugar levels [5]. In addition, the protein–polysaccharide complex, due to its high stability and solubility, can be uniformly dispersed in plant cream, ensuring the consistency and stability of the product. Therefore, protein–polysaccharide complexes have enormous potential for sugar substitution in plant cream. The recent study proved that whey protein [5] and phycocyanin [6] can be used as sugar substitutes in low sugar creams. However, compared with sugar substitutes such as whey protein, PPI has been widely used in food and other fields due to its high nutritional value and low allergenicity [7]. However, the fresh cream prepared from PPI will flocculate or coalesce when the emulsion is stored, mainly due to the collapse of partially coalesced fat spheres and foam structure formed by phase separation. This may be because the O/W interface in the cream structure is easy to break, which promotes the fat spheres to gather together, leading to the instability of emulsion [1]. Therefore, comprehensive control of interface performance is crucial for preparing higher quality fresh cream. Sodium alginate (SA) is a linear anionic copolymer widely used as a food emulsifier. In a mixed system, SA may undergo electrostatic interactions with proteins, resulting in the formation of new structures and textures [8]. Complex structures undergo significant structural unfolding at the oil–water interface [9]. Ultimately, a viscous and viscoelastic interfacial film may be generated to impede the coalescence of oil droplets [10]. Therefore, SA is widely used in protein emulsion food to regulate its structure, function, and stability. In this research, we used the PPI/SA binary complex as a sugar substitute in the cream formulation.

At present, many polysaccharides such as xanthan gum, pectin, locust bean gum, carboxymethyl cellulose, and kappa carrageenan have been used to couple with proteins to stabilize cream [11,12,13]. The addition of these polysaccharides not only further enhances the functional properties of the cream but also improves its sensory attributes. But most research focuses mainly on the evaluation of cream applications. There have been no reports yet on the impact of protein polysaccharide complexes on the properties of fresh cream from the perspective of protein structural differences, interactions between components, and the correlation between O/W interface characteristics. Therefore, it is of great significance to explore the interactions, structural differences, and interfacial behavior mechanisms among the multi-components of PPI/SA, as well as the structure–activity relationship of the macroscopic properties of plant cream. This study chose PPI and SA as research models, established the relationship between their interactions, complex properties, and interfacial behavior, and further investigated the effect of protein–polysaccharide complexes on the quality of plant cream. This provides theoretical support for finding suitable sugar substitute products to develop low sugar plant fat cream and provides new ideas for the production and design of low sugar plant cream, which hold significant practical implications.

## 2. Materials and Methods

### 2.1. Materials

Pea protein (with a protein content of 81.2%) was purchased from Shandong Yuwang Ecological Food Industry Co., Ltd. (Shandong, China). Sodium alginate (purity > 98%), sodium stearate lactate (purity 99%), xanthan gum (purity 99%), and D-sorbitol (purity 99%) were purchased from Beijing Yuanye Biotechnology Co., Ltd. (Beijing, China). We purchased hydrogenated palm oil, white sugar, and sucrose syrup from a supermarket in Harbin.

### 2.2. Preparation of PPI/SA Complexes

Based on the method proposed by Wang et al. [14], we prepared the PPI/SA complexes. Appropriate amounts of PPI and SA powders were dissolved in phosphate-buffered solution (PBS, 0.01 M, pH 7.0) and stirred for 2 h to prepare PPI and SA solutions, with concentrations of 5% (*w*/*v*) and 2% (*w*/*v*) in the PPI and SA solutions, respectively. PPI and SA solutions were mixed in a certain proportion at room temperature to prepare PPI/SA complexes with different mass ratios (1:0, 1:0.1, 1:0.2, 1:0.3, 1:0.4, and 1:0.5). The concentration of PPI in the complex solution is fixed at 1% (*w*/*v*). The pH of all samples was adjusted to 7 using a solution of 0.1 M HCl/NaOH. Table 1 shows the specific preparation formula of PPI/SA complexes.

### 2.3. Characterization of PPI/SA Complexes

#### 2.3.1. Fluorescence Spectroscopy

All samples were diluted to 0.1 mg/mL with PBS and detected by a fluorescence spectrophotometer (F-4500, Hitachi, Japan) at wavelength. The measured parameters included an excitation wavelength of 280 nm and an emission wavelength range of 300–400 nm.

#### 2.3.2. Zeta Potential

We measured the zeta potential of the sample solution using the method by Zhu et al. [8]. Firstly, dilute all samples to 1 mg/mL. Subsequently, we used a 1 mL glass syringe to aspirate the diluted sample solution and slowly injected it into the potential cup until the solution volume reached 2/3 of the potential cup scale. Each sample is scanned 3 times, and the average value is recorded.

#### 2.3.3. Measurement of Flexibility

Protein samples were diluted to 1 mg/mL using phosphate-buffered solution (PBS). Subsequently, 250 μL of trypsin (1 mg/mL) solution was added to 4 mL of the sample solution, and the solution was placed in a water bath at 38 °C for 10 min. Enzymatic hydrolysis was then stopped with 4 mL of trichloroacetic acid (50 mg/mL). Then, the whole solution was spun in a centrifuge at 2500× *g* for 30 min. The amount of digested protein in the clear liquid on top was found out using the BCA method.

#### 2.3.4. Determination of SH Content

Measure the content of free thiols and total thiols according to the measurement method of Hu et al. [15]. Measure its absorbance at 412 nm. Calculate the content of thiol and disulfide bonds according to the following formulas:

$$SH(\mu mol/g)=73.53\times D\times A_{412}$$

$$SS(\mu mol/g)=\frac{{{SH}_{1}-SH}_{2}}{2}$$

Among them, ***D*** represents the dilution factor, and ***A*_412_** represents the absorbance measured at a wavelength of 412 nm.

#### 2.3.5. Circular Dichroism (CD) Spectra

The CD spectra were obtained using a CD spectropolarimeter (JASCO J-810, RI, USA). The wavelength range was set to 190–250 nm for each solution.

#### 2.3.6. Isothermal Titration Calorimetry (ITC)

The ITC experiment was conducted at room temperature using an isothermal titrator (MicroCal Inc., Northampton, UK). All samples should be degassed before the experiment begins [16]. The injection volume was 2 μL, with a total of 25 times and an interval of 350 s. The stirring speed was 250 rpm. A series of thermodynamic parameters were analyzed using NanoAnalyze V01.02.01.01 software.

#### 2.3.7. Interfacial Tension

The interfacial tension analyzer (Sigma 700, Biolin, Sweden) was used to measure interfacial tension. Corn oil was used instead of the fat blend and purified before measuring. If the interfacial tension remained at 29.0 ± 0.5 mN/m for 10,800 s, the corn oil was considered acceptable [17]. The complex solutions of different mass ratios were added into a micro needle sampler, then the needle was inserted into an optical glass colorimetric dish, and 25 μL of the sample was pushed onto the needle tip to form a complete droplet. The CCD camera system captures droplet images at 25 °C for 10,800 s, and the software automatically calculates the interfacial tension value.

#### 2.3.8. Interfacial Protein Concentrations

Interfacial protein concentrations (**Γ**) were ascertained using the previously described method of Yan et al. [18]. A 15 mL sample was transferred into a 50 mL centrifuge tube and subjected to centrifugation at 4 °C and 8000× *g* for 60 min. Subsequently, the aqueous phase and the protein precipitate were gathered. The Kjeldahl method was employed to analyze the protein content. Based on the results, the interfacial protein concentrations could be calculated using the following equation:

$$\Gamma (mg/m^{2})=\frac{V_{c}\times (C_{i}-C_{eq})}{SSA\times V_{oil}}$$

where ***C_i_*** represents the initial protein concentration (mg/mL), ***C_eq_*** represents the concentration of non-adsorbed proteins (mg/mL), ***V_c_*** and ***V_oil_*** represents the volumes of the aqueous and oil phases (mL), respectively, and SSA represents the surface area of the droplets (m^2^/g).

### 2.4. Preparation and Characterization of Whipped Creams

#### 2.4.1. Preparation of Whipped Creams

PPI/SA complex solutions with different mass ratios were created according to the method in 2.2, and then xanthan gum, D-sorbitol, white sugar, and sucrose syrup were added and completely dissolved at 75 °C to obtain the aqueous phase. Hydrogenated palm oil and sodium stearyl lactate (emulsifier) were melted and mixed evenly at a temperature of 75 °C to obtain the oil phase. At 60 °C., the water phase was slowly added to the oil phase while stirring. After mixing the two phases, they were heated at 75 °C for 30 min to ensure thorough and even mixing. The mixture was homogenized at a speed of 8000 rpm for 2 min, then frozen at −18 °C for 12 h, thawed to 4 °C, and stirred until beaten to obtain plant cream. The PPI/SA mass ratio in the prepared plant fat cream is 1:0, 1:0.1, 1:0.2, 1:0.3, 1:0.4, and 1:0.5, with whole sugar plant fat cream as the control. Table 2 shows the formula for plant fat cream.

#### 2.4.2. Determination of Hardness of the Whipped Creams

The hardness of the whipped cream was assessed using texture analyzer (Stable Micro System Co., London, UK). The probe was immersed into the sample at a speed of 3 mm/s to a depth of 15 mm, and the hardness of the whipped cream was determined by the required force (N) [19].

#### 2.4.3. Determination of Overrun of the Whipped Creams

Whipped cream was filled into a 25 mL aluminum dish with dimensions of 40 mm in diameter and 22 mm in depth until reaching full capacity. The weight of the samples was recorded, and the foam overrun was determined using the provided equation. Foam overrun was defined as the ratio of gas weight to liquid weight, expressed as a percentage.

(1) $$Overrun(\%)=\frac{{m_{1}-m}_{2}}{m_{2}}$$

**m_1_**: the mass of unwhipped cream emulsion (g)

**m_2_**: the mass of whipped cream sample (g)

#### 2.4.4. Measurement of Stirring Time and Beating Rate

A proper amount of cream emulsion was taken, and a cream mixer was used to fully stir the cream so that the cream can stand upright as the end of the mixing. The time from the beginning to the end was recorded as the stirring time. The beating rate is the ratio of the mass of the same volume of accumulated water to the mass of the same volume of cream product.

#### 2.4.5. Separation Index of Cream Slurry

According to the method of Van Aken et al. [20] the mass of plant fat cream and the proportion of water droplets in the total mass were calculated to determine the separation coefficient of the cream slurry.

#### 2.4.6. Observation of Stability of Mounting and Stability of Mounting Cutting Surface

The cream was placed in a laminating bag and squeezed into a flower shape through a nozzle to observe the change in flower shape at 0 h and 6 h. After 6 h, the cream was cut to observe the cross-section and photographed for recording.

#### 2.4.7. Apparent Viscosity of the Whipped Creams

The dynamic shear rheometer (DV3THA, Brookfield Corp, USA) was utilized to measure the apparent viscosity of emulsions at 25 °C. The shear rate was systematically increased from 0.1 to 100 s^−1^ over a period of 100 s.

#### 2.4.8. Frequency Sweeps of the Whipped Creams

Using a dynamic shear rheometer (DV3THA, Brookfield Corp, USA), the rheological properties of cream were measured at 25 °C. Frequency sweep testing (1.0–10 Hz) was performed at 0.1% strain to obtain the elastic modulus (G′) and viscous modulus (G″).

#### 2.4.9. Confocal Laser Scanning Microscopy (CLSM)

The cream microstructure was analyzed utilizing CLSM (Zeiss 780, Leica Corp., Germany). After staining, take 5 μL of the sample and place it on a glass slide for measurement and observation.

### 2.5. Statistical Analysis

Each sample was measured three times, and the results were presented in the form of X ± SD. SPSS 19.0 software was used for data analysis, and ANOVA and Duncan tests were used for significant difference comparison. Using software such as Origin 2022b, Paekfit 4.12, Adobe Illustrator 2020 for data processing and chart production.

## 3. Results

### 3.1. Fluorescence Spectroscopy

**Figure 1:**
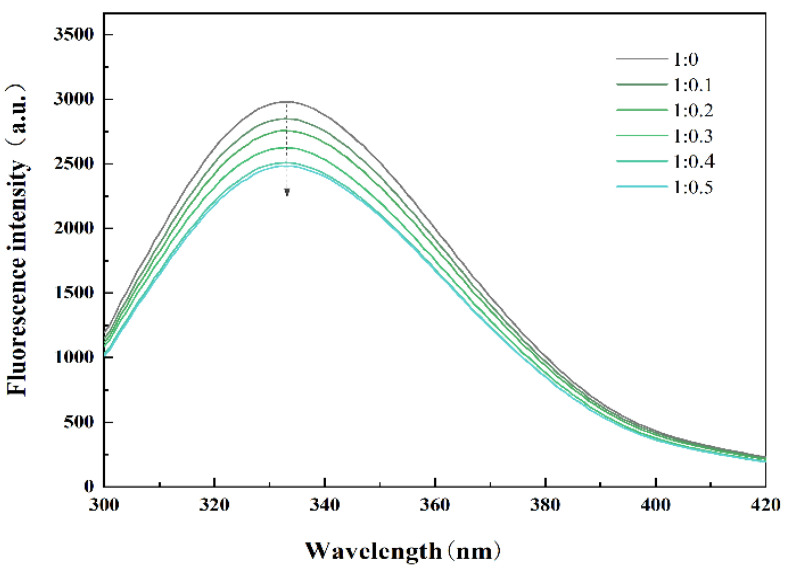
Fluorescence spectrum of PPI/SA complexes with different mass ratios.

Usually, the fluorescence spectrum of proteins is due to the presence of aromatic amino acids, whose positions largely depend on the degree of protein folding and can be used as a sensitive monitor for protein conformational changes [21]. In addition, the change in protein tertiary structure will affect the migration rate of protein to the oil–water interface and the ability to form an interfacial film, thus, affecting the quality of cream. Figure 1 shows the fluorescence spectra of PPI and PPI/SA complexes. The results indicate that a gradual decrease in peak fluorescence emission intensity of PPI at 333 nm is observed as the concentration of SA increases. This indicates that the interaction between sodium alginate and PPI occurs near the aromatic amino acid region, and the aromatic amino acid groups in the protein structure are buried by sodium alginate molecules. In addition, the addition of SA can cause a change in the spatial conformation of PPI [22]. It is generally believed that folded proteins exhibit higher fluorescence intensity (FI), and partial or complete unfolding of proteins can lead to a decrease in FI. The experimental results indicate that the addition of SA leads to the unfolding of PPI and alters the spatial configuration of amino acids within the protein and exposes previously shielded hydrophobic amino acids to a hydrophilic polar environment [23]. This unfolded structural feature will promote the reduction in interfacial tension, accelerate the migration rate of PPI from the water phase to the oil drop surface, and, thus, improve the ability of protein to form interfacial film.

### 3.2. Zeta Potential

**Figure 2:**
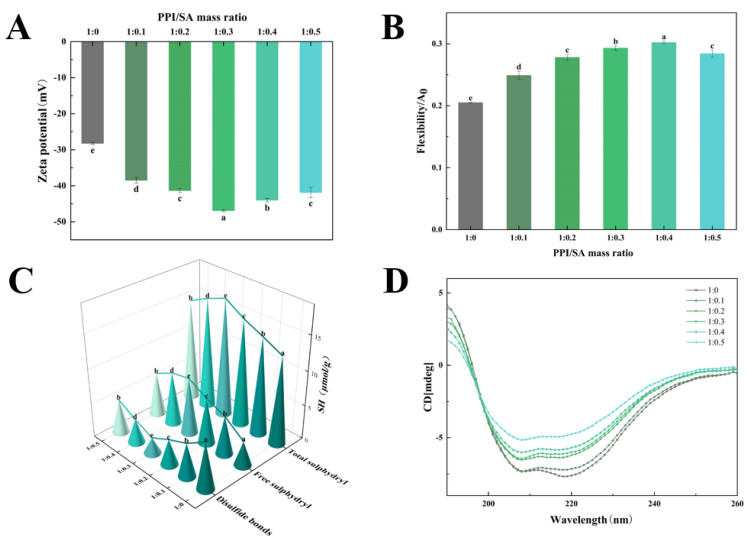
Physicochemical and structural characterization of PPI/SA complexes: zeta potential (**A**), protein flexibility (**B**), sulfhydryl and disulfide bond content (**C**), and CD spectra (**D**). Note: Different letters indicate significant differences (*p* < 0.05) between sample mean values, the same applies below.

The Zeta potential serves as a crucial indicator for assessing system stability. The Zeta potential values higher than ±30 mV suggest that the electrostatic repulsions between molecules are sufficiently strong [24]. Figure 2A shows the Zeta potential of PPI/SA complexes with different mass ratios. Compared to PPI, when SA is present in the solution system, the absolute value of the Zeta potential increases. As the concentration of SA increases, the absolute value of the Zeta potential of PPI exhibited an initial increase and subsequent decline. And the absolute value of the Zeta potential reaches the maximum value when the PPI/SA mass ratio is 1:0.3. Firstly, the increase in Zeta potential may be due to the exposure of hydrophilic polar groups. The functional groups on SA can undergo hydrogen bonding or hydrophobic interactions with proteins, leading to structural changes in proteins and promoting the exposure of hydrophilic groups. Secondly, as SA is a natural anionic polysaccharide, it can attach to the surface of PPI and enhance its negative charge. When the mass ratio of PPI/SA exceeds about 1:0.3, the positive charge on the PPI is saturated with SA, and any excess SA may promote the aggregation of the complex through a depletion mechanism. This may lead to excessive SA increasing the coverage of the protein surface and exerting a shielding effect, thereby reducing the absolute value of the negative Zeta potential.

### 3.3. Protein Flexibility

The flexibility of proteins influences the adsorption and rearrangement kinetics of proteins at the interface, consequently impacting the stability of protein-based creams at the interface [25]. Figure 2B shows the molecular flexibility of PPI/SA composites. As concentration of SA increases, the flexibility of proteins first increases and then decreases compared with natural PPI (*p* < 0.05). When the mass ratio of PPI/SA complex is 1:0.4, the highest molecular flexibility occurs. The increased flexibility of the complexes may be due to the interaction between PPI and SA, which leads to a certain degree of change in the distribution of amino acid residues in the main peptide chain of PPI. This promotes the dissociation of protein subunits and changes in secondary structure. The dissociation of subunits will gradually expand the conformation of the protein, exposing the non-polar residues previously buried in the protein, and the protein will transition from an ordered structure to a disordered structure [26]. These lead to an increase in the flexibility of the PPI structure. PPI with higher flexibility can quickly adsorb and rearrange at the oil–water interface, thereby reducing interfacial tension and enhancing emulsifying performance. When the mass ratio of PPI/SA complex is 1:0.5, the molecular flexibility of PPI decreases. This may be due to the excessive reaction between SA and PPI, which generates strong electrostatic repulsion and steric hindrance, inhibiting the expansion and extension of PPI molecules, thereby reducing their flexibility [27]. This phenomenon will result in a decrease in the diffusion rate of PPI towards the oil–water interface, hindering the adsorption of proteins at said interface.

### 3.4. Free Sulfhydryl Content

Sulfhydryl and disulfide bonds are important chemical bonds that maintain the spatial structure of proteins and endow them with certain functional properties, and their content is dynamically changing [28]. Figure 2C shows the sulfhydryl groups and disulfide bond contents of PPI/SA complexes with different mass ratios. The results indicate that as the concentration of SA increased, the total sulfhydryl groups content and free sulfhydryl groups content first increased and then decreased, while the disulfide bond content showed the opposite change. When the mass ratio of PPI/SA complex is 1:0.3, the total sulfhydryl groups and free sulfhydryl groups content reaches its peak, and the disulfide bond content reaches its lowest value, which is 27.84 μmol/g, 0.55 μmol/g and 6.74 μmol/g, respectively. The reason for this phenomenon may be due to the interaction between PPI and SA, which leads to changes in protein structure and subunit dissociation, promoting the exposure of -SH groups and the breaking of disulfide bonds in proteins [29]. However, when the mass ratio of PPI/SA complexes exceeds 1:0.3, the content of disulfide bonds increases, while the total sulfhydryl groups content and free sulfhydryl groups content decrease. This may be due to the formation of larger particle size aggregates in the presence of high concentrations of SA, which weakens the interaction between SA and PPI. This is not conducive to the complete unfolding of protein and the exposure of free sulfhydryl groups and leads to the transformation of free thiol groups into disulfide bonds [30].

### 3.5. Secondary Structure

The changes in the secondary structure of proteins can affect their interfacial properties to a certain extent, so CD spectroscopy is used to analyze changes in the secondary structure of PPI/SA [31]. Figure 2D shows the CD spectroscopy results of PPI/SA complexes. As the SA concentration gradually increases, the maximum negative average residual ellipticity of PPI/SA complexes also significantly increases. Meanwhile, within the wavelength range of 213 to 230 nm, the negative ellipticity of the shoulder peak also increases, indicating a change in the secondary structural conformation. Table 3 shows the secondary structure content of proteins. As the SA concentration gradually increases, the β-sheet content increased from 13.87% to 25.02%, while α-helix content decreased from 24.83% to 17.72%. At the same time, the content of random coil has decreased. The α-helix of protein is maintained by intra-chain hydrogen bonds. The decrease in the content of α-helix indicated that the interaction between PPI and SA led to the break of intra-chain hydrogen bonds and the formation of interchain hydrogen bonds [32]. This is also the reason for the increase in β-sheet content. This transformation indicates that during the unfolding process of PPI, the deeply masked intramolecular hydrogen bonds within the protein may be disrupted. A lower α-helix content and increased flexibility of a more disordered structure may accelerate the protein rearrangement at the oil–water interface, thus, improving the stability and functionality of the emulsion [33].

### 3.6. ITC

**Figure 3:**
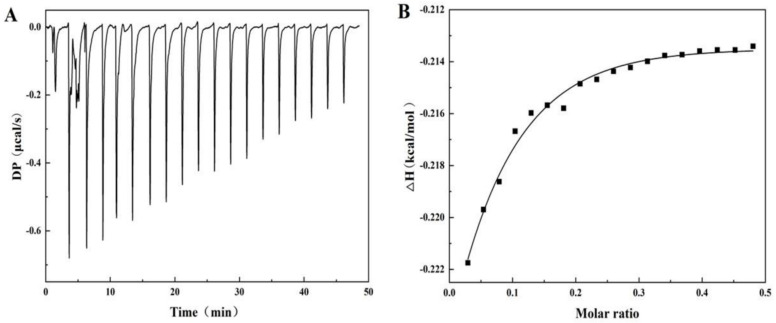
The curve of heat flow vs. time (**A**) and corresponding binding isotherms (**B**) during the titration of PPI (0.29 mM) with SA (0.02 mM) in phosphate-buffer solution (10 mM, pH 7.0).

Isothermal titration calorimetry (ITC) is a method of qualitatively characterizing protein/ligand interactions through thermodynamics. The main advantage of ITC is that it can sensitively measure the thermal changes caused by intermolecular interactions [34]. Figure 3A shows the thermal spectrum of the heat flow rate and time when titrating 40 mg/mL SA to PPI. Obviously, each peak corresponds to the thermal changes associated with the calorimeter unit when injecting SA solution equally into the PPI solution. The heat flow curve shows a negative heat flow change, indicating that the entire process is a combination of exothermic processes. This means that the binding between SA and PPI is beneficial for the reduction in system energy [34]. In addition, the heat flow will gradually decrease with the increase in SA titration amount and eventually remain stable, indicating that the sites on the surface of PPI gradually decrease with the binding of SA and eventually reach saturation. The experiment obtained thermodynamic binding parameters (Figure 3B) by fitting the titration curve using a unit point independent binding model, such as binding stoichiometry (N), binding constant (Ka), enthalpy change (ΔH), and entropy change (ΔS) [35]. The binding ration of the reaction was calculated to be 1.56 (± 12.8), indicating that each PPI molecule can bind approximately 1.56 SA monomer molecules, further indicating the formation of a complex between PPI and SA. The interaction between two molecules with a binding constant, Ka, greater than 104 M^−1^ is considered to have a high affinity and can undergo strong interactions. The Ka value measured in this experiment is 2.72 ± 0.32 × 103 M^−1^, indicating a non-specific interaction and relatively weak binding between PPI and SA. The values of TΔS, ΔH, and ΔG are 3.28, −0.218, and −3.5 kcal/mol, respectively, indicating that PPI/SA binding is a mainly entropy driven exothermic process. Therefore, current results indicate that SA mainly binds to the hydrophobic region of PPI through non-specific interactions, which is consistent with results of fluorescence spectroscopy [36]. Similar behaviors also occur in the interaction between seaweed lipid biosurfactants and BSA [37].

### 3.7. Adsorption Kinetics of Protein/Emulsifier Mixture

**Figure 4:**
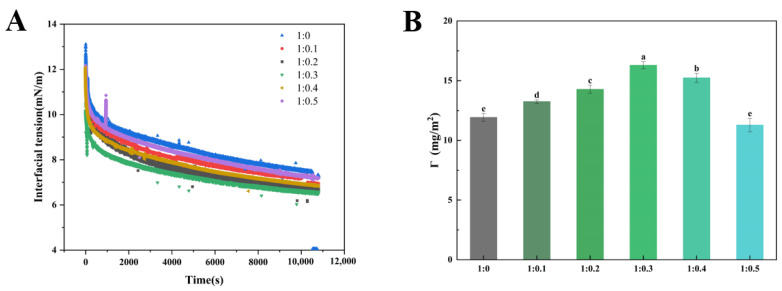
Interfacial tension (**A**) and protein concentration (**B**) at different mass ratios of PPI/SA (**B**). The letters indicate significant differences (*p* < 0.05).

Whipped cream is a typical O/W cloudy system before whipped, so the interfacial tension of solution is crucial to the stability of cream [38]. In order to investigate the effect of the adsorption kinetics of proteins and polysaccharides at the oil–water interface on the stability of aerated cream, we measured the interfacial tension changes in PPI/SA complexes at different mass ratios. As shown in Figure 4A, the characteristic of interfacial tension change was a rapid decrease at first, followed by a slowdown in the rate of reduction over time and eventually reaching equilibrium. This is related to the different stages of PPI adsorption (diffusion, penetration, unfolding, and rearrangement) [39]. Driven by external energy, PPI can quickly adsorb to the oil–water interface, decreasing thermodynamic unfavorable contact between different types of molecules at the interface and reconfiguring themselves to reduce interfacial tension and forming a protective interfacial film through intermolecular interactions [40]. Throughout the entire time frame, the interfacial tension of PPI and SA composite is lower than that of PPI alone. These findings indicate that SA may coexist with proteins at the oil–water interface in a cooperative manner to promote a decrease in interfacial tension. The presence of sodium alginate can greatly affect interfacial tension through a competitive adsorption with proteins and/or polysaccharide–protein complexation effects. Moreover, polysaccharides can access the protein stabilized interface via small gaps in the entangled protein layer, leading to decreased interfacial tension [41]. However, when the mass ratio of PPI to SA exceeds 1:0.3, the interfacial tension of the complex increases. This may be due to the excessive accumulation of polysaccharides on the hydrophobic side chains of proteins after their binding, which limits the adsorption and rearrangement of proteins at the oil–water interface. This has a negative impact on the diffusion of PPI/SA complex from bulk phase to interface. Secondly, it may be that the protein on the oil–water interface film will be replaced by too much SA, which will lead to instability of emulsion and cream [42].

### 3.8. Interfacial Protein Concentration

Interfacial protein concentration refers to the protein content per unit area, which is the reflection of protein adsorption at the O/W interface in the emulsion system [43]. Figure 4B shows that the interface protein concentration varies among different samples. As the concentration of SA increases, the interface protein concentration shows a trend of first increasing and then decreasing. When the mass ratio of PPI/SA complex is 1:0.3, the interface protein concentration reaches its peak. The underlying cause of the above—mentioned phenomenon lies in the fact that the hydrophobic groups of the protein possess the ability to break free from the poor solvent. As a result, they tend to spontaneously adsorb at the oil—water interface. The significant increase in interface protein concentration may be partially attributed to the increase in spontaneous protein adsorption. In addition, when PPI and SA coexist, the interfacial synergistic/competitive adsorption behavior usually occurs on the interface membrane. When the amount of SA added is low, PPI dominates at the interface, and SA can adsorb on the surface and gaps of proteins. This leads to an increase in thermodynamic incompatibility: the adsorbed PPI is concentrated by polysaccharides, thereby accelerating the diffusion and adsorption of PPI at the interface. Generally, a higher quantity of protein present at the oil—water interface was more conducive to the formation of a thicker interfacial protein layer that could enwrap the oil droplets to achieve the emulsion stability [44]. As the concentration of SA gradually increases, excessive SA disrupts the protein–protein interactions at the oil–water interface. This leads to PPI being replaced and desorbed by SA from the surface of fat globules to the continuous phase [45]. The interface film with polysaccharides or aggregates as the main body was slowly formed, leading to a decrease in the concentration of interface proteins. The decrease in interfacial protein concentration will reduce the strength of lotion interfacial film, which promotes the formation of partial coalescence structure of fat, leading to the decline of cream quality [46]. The above research results confirm the importance of different SA addition amounts in enhancing the affinity of PPI to the oil–water interface, thereby helping to improve the stability of cream.

### 3.9. Texture Analysis of Cream

**Figure 5:**
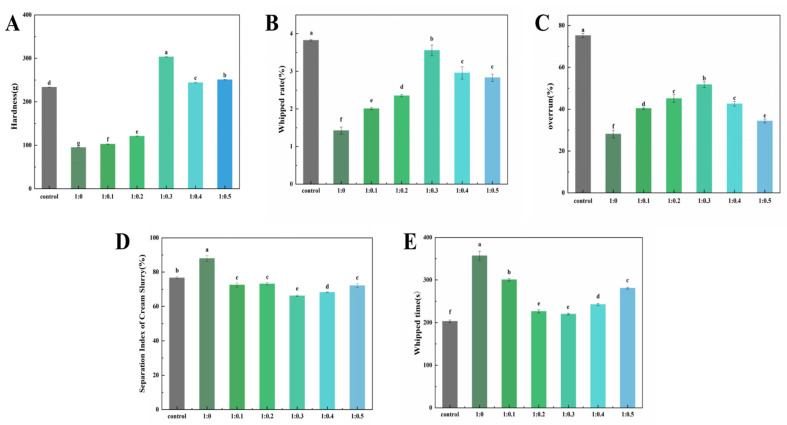
The effect of PPI/SA complex mass ratio on the hardness (**A**), whipped rate (**B**), overrun (**C**), separation index (**D**), and whipped time of whipped cream (**E**). The letters indicate significant differences (*p* < 0.05).

Hardness refers to the maximum pressure applied to cream to initiate deformation and plays a crucial role in determining the processing characteristics and edible quality of cream products [47]. It can be utilized to assess the capacity of plant-based cream to retain foam structure and stability after whipped, thereby influencing the ultimate acceptance of the cream. The hardness of cream with different mass ratios of PPI/SA complexes replacing sugars and whole sugar cream is shown in Figure 5A. As the concentration of SA increases, the hardness value of cream first increases and then decreases. Among them, the hardness value of the 1:0.3 sample is the highest (303.40 ± 0.88). This might be due to the fact that SA makes PPI stretch and reveals some hydrophobic groups. Consequently, its binding ability to fat globules is enhanced. This will promote the movement of fat globules towards the surface of bubbles and further aggregate to create a dense network structure, thereby improving the anti-deformation ability of the cream [48]. On the other hand, PPI and SA will be adsorbed on the surface of the fat sphere to form a hard oil–water interface film. The PPI/SA complex helps to form a thicker facial mask interface, thus, improving the hardness of whipped cream. From the results, it can be seen that the overall improvement of SA on the hardness of fresh cream indicates that SA is effective in changing the quality of fresh cream [49]. When the proportion of SA continues to increase, the hardness value of cream significantly decreases (*p* < 0.05), which may be due to the excessive aggregation of large protein aggregates induced by excessive SA during the stirring process, rather than partial aggregation. The strength of the oil–water interfacial film structure formed by excessive coalescence is weaker than that of the oil–water interfacial film structure formed by partial coalescence, so the network structure formed is looser and has lower hardness.

### 3.10. Analysis of Cream Whipped Rate

The whipped rate refers to the ability of cream to wrap air during whipping, and its size is an important performance index reflecting the quality of whipped cream. The cream with higher whipped rate can obtain good foam hardness, luster, fineness, and instant melting in the mouth [50]. The effect of the PPI/SA complex mass ratio on the whipped rate of whipped plant fat cream is shown in Figure 5B. As the addition of SA increases, the whipped rate first increases and then decreases. When the PPI/SA is 1:0.3, the whipped rate is closest to the control sample. This may be due to the fact that as the concentration of SA increases, the continuous network formed in the continuous phase can effectively suppress the collision frequency of fat globules, resulting in a gradual increase in whipped rate. When the amount of SA added is too high, the beating rate decreases. The main reason is that the increase in SA addition leads to an increase in the apparent viscosity of the system, making it difficult to quickly fill air during the stirring process and resulting in an increase in bubble rupture rate. Secondly, when the amount of SA added is too high, the concentration of liquid phase protein decreases, and the volume of air filled in the first stage of stirring decreases. The lower part of the aggregation rate is not conducive to wrapping bubbles and forming fat network structures [51].

### 3.11. Analysis of Cream Overrun

Overrun rate is an indicator that provides information on the gas content or gas percentage in fresh cream. It is an important parameter of foam food, because it affects its texture, taste, stability, and overall quality. Cream with a higher overrun rate typically has a lighter and more fluffy texture [52]. As shown in Figure 5C, as the concentration of SA increases, the overrun rate of the sample first increases and then decreases. In the whipped cream system, the partial coalescence of fat globules in the oil phase contributes to the formation of a network structure of fat crystals. Meanwhile, a high oil phase mass fraction promotes the formation of a network structure of fat crystals, thereby accelerating the partial coalescence of fat globules [53]. In contrast, the overrun rate of protein samples is only 28.15%. This is because the coalescence speed of fat globules is very slow, and they cannot quickly form a mesh structure to wrap around enough air. After adding SA, the overrun rate significantly increased, which may be because SA can interact with PPI to form aggregates. The cream network structure is formed through aggregation, and the aggregated fat ball can have a stronger skeleton, which makes the foam more concentrated. However, as the concentration of SA increases, the degree of protein aggregation becomes too high, resulting in uneven protein distribution and a lack of continuous three-dimensional network structure [54]. In addition, its rapidly forming network structure resulted in the encapsulation of less air. Nooshkam et al. [5] confirmed that the ternary compound bioactive foam can partially replace the fat and sugar in ice cream, while maintaining a high overrun. These results show that an appropriate amount of SA can rapidly stabilize on the surface of AW to promote the formation of foam. The synergistic effect of PPI and SA promotes the aggregation/coalescence of fat globules, encapsulating sufficient air.

### 3.12. Analysis of Separation Index of Cream Slurry

The slurry separation index of cream is a parameter that describes the degree of slurry separation in liquid mixtures (cream) [20]. The degree of separation can affect the quality, taste, and appearance of cream. For cream, a lower slurry separation coefficient is usually ideal. Because cream with a lower slurry separation coefficient can maintain a uniform texture and is less prone to significant phase separation and structural changes. The effect of the PPI/SA complex mass ratio on the separation index of whipped plant fat cream slurry is shown in Figure 5D. Cream prepared from pure protein samples quickly leaks out of the sieve, resulting in a high separation index of the slurry. As the amount of SA increases, the slurry separation index of the sample shows a trend of first decreasing and then increasing. This may be due to the fact that with the addition of SA, the cream will soon enter the stable agglomeration stage after being stirred. At this time, the hardness and viscosity of the cream will increase, and the bubbles will start to form stable foam. The whipped cream at this stage can be considered as a system with very small and tightly arranged bubbles, and a complex is adsorbed on the surface of the bubbles, in a stable state. The results show that SA plays a key role in stabilizing bubbles, and PPI/SA complex can stabilize the microstructure of liquid foam in low sugar system. When the amount of SA added is too high, the separation coefficient of the slurry shows an upward trend. At this time, stirring will cause excessive agglomeration of fat globules, resulting in a rapid decrease in foaming rate during stirring [55]. The agglomerates also begin to form larger aggregates, leading to an increase in the separation coefficient of the slurry. This leads to the inability of plant fat creams to form a continuous network and effectively capture water, resulting in an increase in the slurry separation coefficient [56].

### 3.13. Analysis of Cream Whipped Time

The whipping time refers to the time required to whip the cream into a normal shape. A shorter whipped time indicates that the whipped cream system can be filled by air faster, thereby maintaining the stability of the system [57]. Therefore, whipped time is one of the important indicators for estimating the whipping performance of cream. The effect of the PPI/SA complex mass ratio on the whipped time of plant fat cream is shown in Figure 5E. The PPI sample requires about 6 min or even longer whipping time to reach the whipped endpoint. This is because the PPI structure is loose and cannot form a strong boundary film, and the system viscosity is small, which leads to easy leakage of bubbles and requires a long whipping time. Especially when only protein is present in the alternative system, the large fat globules formed by excessive aggregation will destroy the bubble structure, resulting in oil separation and defoaming phenomenon. As the concentration of SA increases, the whipping time of the sample first decreases and then increases, with the shortest whipping time occurring at 1:0.3. This is because increasing the proportion of SA appropriately can ensure sufficient gas entry and promote partial aggregation of fat globules, thereby quickly forming a three-dimensional network structure that wraps around the captured bubbles [58]. Secondly, the hydrophilic/hydrophobic structure of SA makes it easier to adsorb at the oil–water interface and reduces the surface tension of the liquid phase. An appropriate amount of SA can provide PPI/SA complexes with more flexible interface behavior, faster adsorption rate and higher rearrangement efficiency. This leads to an improved resistance to crystal puncture at the interface. Finally, SA is an effective surfactant that can replace proteins at the interface, leading to partial crystallization of lipids. When the amount of SA is low, proteins dominate the interfacial properties. However, due to its high interfacial activity, SA can replace proteins and reduce steric hindrance between droplets. This is beneficial for the flow of liquid fat, accelerating fat crystallization and shortening whipped time. As the SA content continues to increase, the whipped time begins to increase. This is mainly because excessive SA increases the viscosity of the system, making it difficult for bubbles to enter the cream system [59]. This makes it difficult for cream to wrap bubbles, making it easier for fat balls to come into contact with each other and aggregate, thereby increasing whipping time. In addition, when the concentration of SA is high enough to replace the main interface properties of protein, the viscoelasticity of the interface film will increase instead [60]. This will prevent the aggregation of fat to some extent, so the whipping time will be extended. Camacho et al. [61] also found that increasing the viscosity of cream will promote the rapid development of elastic properties of whipped products. That is to say, after adding less air, foam will be quickly separated from the mixer, reaching the air limit.

### 3.14. Observation and Analysis of the Appearance and Section of Mounted Flowers

**Figure 6:**
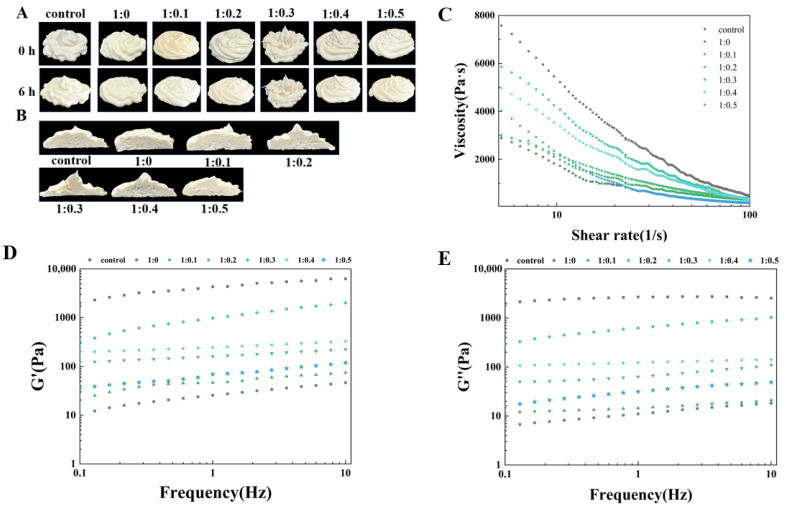
The effect of PPI/SA complex mass ratio on the decorating stability (**A**,**B**), apparent viscosity (**C**), and elastic/viscous modulus (G′ and G″) (**D**,**E**) of whipped cream.

Fresh cream is typically consumed within a brief time, making short-term storage stability a crucial characteristic of the product. The storage stability of cream produced from PPI/SA complexes with varying proportions can be assessed through observing alterations in their rose-shaped morphology and extent of collapse over different storage durations [62]. Figure 6A displays the visual appearance of cream at varying storage times (0 h, 6 h), while Figure 6B illustrates the roughness of the cream cross-section. It can be observed that the control sample has sharper edges, stronger peaks, clearer textures, and no surface bubbles. On the contrary, PPI samples cannot exhibit a rose-like structure due to their rapid collapse, resulting in a rough surface and the generation of many bubbles. After adding SA, the cream stabilized by PPI/SA complexes initially exhibited a rose-like appearance, with clear textures and firm peaks. With an increase in the storage time of cream, the storage stability of all cream samples decreases. This is due to the consumption of bubbles (drainage, coalescence, and disproportionation) leading to varying degrees of collapse. Compared to other samples, when the mass ratio of PPI/SA is 1:0.3, the degree of cream collapse is lower. It can be clearly seen from the figure that the peak height and overall appearance of the shape of the sample stored for 6 h have not changed much compared to the shape at 0 h. This indicates that a PPI/SA complex mass ratio of 1:0.3 is more conducive to improving the storage stability of cream in the cream system [63]. After adding SA, the appearance of the cream cross-section also showed a similar trend. The pore distribution inside the cream is more uniform, and the cross-section is smoother. Among them, when the mass ratio of PPI/SA is 1:0.3, the sample has the most uniform pore distribution and smoothest cross-section. The main reason for this phenomenon is that when an appropriate amount of SA acts as a bridge between PPI and protein aggregation, a three-dimensional network may be formed. By forming these three-dimensional networks, fat balls wrapped in bubbles are inhibited from freely flowing and aggregating, resulting in uniform pores distribution. At the same time, it improves the stability of foam by providing a solid framework [64]. When the proportion of SA continues to increase (PPI/SA is 1:0.5), the cream becomes less stable, and its smoothness decreases as well, with pores that are larger and unevenly distributed. It may be due to the PPI aggregates that form at high SA concentrations that hinder network continuity. This resulted in uneven holes on the cross-section of the cream.

### 3.15. Analysis of Cream Whipped Time

The apparent viscosity of cream is also an important indicator for evaluating its whipped performance, texture quality, and acceptability. According to general theory, an increase in the apparent viscosity of cream leads to a decrease in the migration of fat globules. Reducing the chance of collisions between fat globules helps maintain the long-term storage stability of whipped cream [65].

The effect of PPI/SA complexes mass ratio on the apparent viscosity of whipped plant cream is shown in Figure 6C. All samples exhibit a trend of shear thinning, where the apparent viscosity gradually decreases with an increasing shear rate. This is because plant cream is a pseudo plastic fluid, where shear action disrupts or recombines the internal network structure of plant cream, resulting in a decrease in apparent viscosity. As the amount of SA increases, the apparent viscosity of cream appears to increase and then decreases. When PPI/SA is 1:0.3, the apparent viscosity of cream is the highest. This may be due to the interaction between SA and PPI, which facilitates the binding between droplets and inhibits the flow of cream. When PPI and SA are combined in the cream system, the droplets will be closer together, resulting in increased flow resistance between the droplets. This leads to an increase in the apparent viscosity of the cream. The reason may be that the addition of SA can promote the unfolding of protein structure, thereby enhancing the ability to adsorb at the oil–water interface and stabilizing small droplets through tight arrangement [66]. This changes the enhanced droplet–droplet interaction per unit volume, thereby reducing droplet fluidity. This will make the cream more resistant to damage under the same shear force, leading to higher apparent viscosity. However, when the amount of SA is too high, the apparent viscosity decreases. This may be because excessive SA may cause the droplet structure to become disorganized, thereby increasing voids and reducing the degree of arrangement tightness. Therefore, when excessive SA is added, the degree of polymerization between droplet structures decreases, weakening the formed three-dimensional network structure and resulting in lower apparent viscosity [67].

### 3.16. Analysis of Frequency Scanning of Cream

Frequency scanning can be further used to investigate the effect of PPI/SA complex mass ratio on the internal structural stability of whipped plant cream. The elastic modulus (G′) reflects the ability of cream to maintain its shape and resist deformation during storage. The viscosity modulus (G″) reflects the viscosity of cream. The elastic modulus and viscosity modulus of cream are influenced by the internal network structure and intermolecular interactions of cream [68]. The effect of the PPI/SA composite mass ratio on the viscosity/elastic modulus of whipped plant cream is shown in Figure 6D,E. In the whole frequency range (0–10 Hz), all creams exhibit a slight increase in their elastic modulus and viscous modulus as shear frequency increases. Furthermore, it is observed that the elastic modulus of all cream samples surpasses the corresponding viscosity modulus. The G’ and G″ of the control cream sample are higher than those of the pure protein sample. This indicates that the internal network structure strength of the control sample is stronger than that of the pure protein sample. As SA increases, the G′ and G″ of cream first increase and then decrease, reaching their maximum value when PPI/SA is 1:0.3. It can be inferred that at appropriate concentrations, SA acts as a bridge to further stabilize the three-dimensional network structure formed by the aggregation of PPI. Therefore, a double stable strong network structure is formed inside the cream, which improves the viscosity and elasticity of the cream and enhances the stability of the internal structure of the cream [68]. However, when the amount of SA is too high, the formed macromolecular aggregates may weaken the interaction between SA and PPI, reducing the viscoelasticity of cream. Reference [30] also reported similar behaviors of foam food.

### 3.17. CLSM

**Figure 7:**
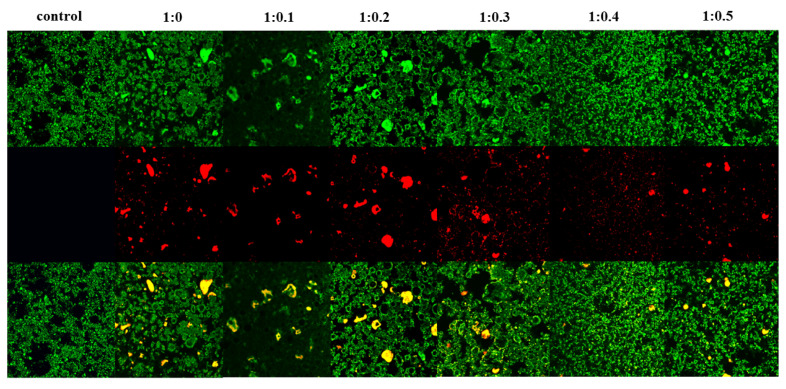
The CLSM images of whipped cream.

Both protein distribution and fat globule aggregation influence stability of whipped cream [54]. Figure 7 displays the microstructure of whipped cream as observed through CLSM, illustrating the red fluorescence of proteins and the green fluorescence of fats. Furthermore, black holes are air bubbles surrounded by protein and fat globules. At the same level of magnification, it was noted that the control sample exhibited numerous densely packed small air bubbles in close proximity to one another. The observed phenomenon suggests an increased level of network formation among air bubbles, thereby enhancing the structural integrity of the foam. When PPI/SA is 1:0, the air bubbles in the cream were wrapped by protein in liquid form, exhibiting irregular aggregation and flocculation. The observed deformation of the bubbles suggests a deficiency in foam stability [69]. When SA was incorporated, the micrographs showed air bubbles with a more homogenous and spherical form. Meanwhile, the fat globule aggregates, and proteins were uniformly arranged, which led to the construction of a network. According to the findings presented above, it is evident that the inclusion of SA enhances the protein aggregation and reduces excessive fat accumulation within the foam structure. This is beneficial for forming a stable bubble network [70]. Among them, when PPI/SA is 1:0.3, the surface of the bubbles covered by the complexes is completer and denser, forming a clear protein interface layer. One possible explanation is that the presence of SA may lead to conformational alterations and molecular unfolding of PPI, thereby exposing hydrophobic and hydrophilic residues. These exposed hydrophilic and hydrophobic residues can cross the air–liquid interface, which promotes the adsorption, expansion, and reorganization of the PPI/SA complex on the interface, thus, establishing a compact interface layer and improving the stability of foam. However, no such variability was observed in sample with PPI/SA of 1:0.3. This could be attributed to SA enhancing the viscosity of the foam liquid film’s surface, thereby impeding the liquid’s flow within the film. Specifically, the small and compact bubbles offer a significant level of resistance to deformation and serve to maintain the structural integrity of the foam. In addition, the distribution of free complexes in cream is relatively uniform. Han et al. [71] have confirmed that the number of complexes that can be adsorbed onto the interface is limited, which means that some complexes particles will be free as non-adsorbed particles in the cream system. These non-adsorbed complexes may tend to produce three-dimensional network structures, which also help stabilize foam in cream. However, when the addition of SA is too high, the image shows agglomeration and an increase in particle size, indicating the formation of irregularly shaped large aggregates. Extensive protein adhesion and excessive aggregation of fat globules were observed within the foam structure. It is evident that the quantity of total protein and fat globules in whipped cream remains consistent. A high concentration of SA treatment leads to the re-aggregation of PPI, forming large protein aggregates. When a large amount of protein and fat clusters gather in a relatively small area, it is likely that there will be a shortage of bubbles, stabilizing substances in other areas [72].

### 3.18. Correlation Analysis

**Figure 8:**
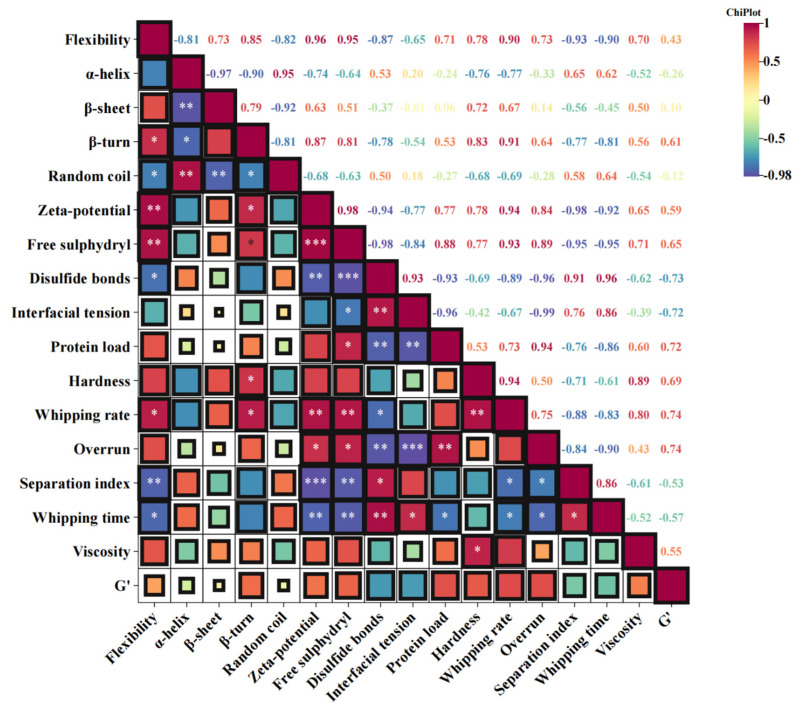
Correlation map between various indicators of PPI/SA complexes and creams. Note: * represents *p* < 0.05, ** represents *p* < 0.01, *** represents *p* < 0.001.

The relevant analysis figure depicts Pearson’s correlation coefficient (r), ranging from −1 to 1, and uses color codes to enhance understanding of the degree of correlation, as shown in Figure 8 [73]. It can be seen from the figure that the structural change in protein (flexibility, potential, and free sulfhydryl group) is negatively related to the interfacial tension of the complexes and is positively related to the interfacial protein concentration of the emulsion, the viscosity, and the storage modulus of the cream. The results indicate that after adding SA, PPI has stronger flexibility and higher potential, which is more conducive to obtaining complexes with better interface performance and stability. The augmentation of electrostatic repulsion is advantageous for promoting a more homogeneous and compact adsorption of PPI at the interface, whereas the reduction in interfacial tension will enhance the rate of PPI adsorption at the interface [65]. Meanwhile, the interfacial tension of composite materials is positively correlated with the hardness of cream, beating rate, and exceeding the limit, while negatively correlated with separation coefficient and stirring time. This indicates that the better the interfacial performance of PPI/SA complexes, the better the stirring performance, texture, and stability of cream prepared as a sugar substitute. During the interaction between PPI and SA, proteins undergo unfolding and structural rearrangement, causing structural changes and subunit dissociation, resulting in exposure of internal -SH groups and disulfide bond cleavage. When the SA content is moderate, SA forms a more complex and robust molecular network in the cream system, which is less susceptible to shear damage and ensures the safe entry of trapped bubbles. This enhances the stability of cream foam, which will promote cream to form a stronger network structure and better wrap the air [74]. In addition, the fine crystals of SA easily pierce the interface film between fat spheres during collision, forming crystal bridges with adjacent crystals and quickly forming a coalescence network of fat parts. This enhances the texture characteristics of cream. When the content of SA is high, excessive polymerization of cream can lead to an incomplete or loose network structure, making it difficult for bubbles to maintain stability [75]. Therefore, the different ratios of PPI and SA have a certain impact on the internal structure of the cream, which in turn affects the quality and performance of the cream.

## 4. Conclusions

This study demonstrated the feasibility of using PPI/SA complexes instead of sugar in low sugar creams by studying the structure and interface properties of PPI/SA complexes with different mass ratios, as well as the influence of PPI/SA complexes on cream properties. Systematically explored the effects and regulatory mechanisms of PPI/SA complexes with different ratios in replacing sugars in plant cream. SA alters the physical and chemical properties of protein molecules, such as flexibility and intermolecular interactions, by affecting the electrostatic repulsion, steric hindrance, and structure of PPI. When the ratio of PPI/SA is 1:0.3, the mixing performance and quality of plant cream are best, including drainage rate, beating rate, mixing time, and slurry separation index. The bridging effect of SA enhances the interaction between PPI and SA, endowing the interface layer with higher rigidity and flexibility. The strong interface characteristics enable it to quickly and accurately respond to external deformation, effectively prevent interface cracking due to stress, and ensure the long-term stability of the cream system at the macro level. As the concentration of SA further increases, the degree of fat partial aggregation decreases, and the formed partial aggregation network structure is incomplete, leading to a decrease in the overall performance of the cream system. This study elucidates the correlation between the structure, interaction, interface properties, and basic properties of PPI/SA complexes. The conclusions of this study provide valuable theoretical support and reference for the research, production and application of functional properties of emulsion food.

## Figures and Tables

**Table 1:** The Specific preparation formula of PPI/SA complexes.

PPI/SA Mass Ratios	5% PPI/ml	2% SA/mL	PBS/mL
1:0	20	0	80
1:0.1	20	5	75
1:0.2	20	10	70
1:0.3	20	15	65
1:0.4	20	20	60
1:0.5	20	25	55

**Table 2:** The formula for plant fat cream.

Component	Content
D-sorbitol	0.95%
White sugar	7.51%
Sucrose syrup	15.02%
Xanthan gum	0.81%
Hydrogenated palm oil	25%
Sodium stearoyl lactylate	1.08%
PPI/SA complexes	Add according to experimental requirements
Water	Supplement to 100%

**Table 3:** The secondary structure content of PPI/SA complexes with different mass ratios.

PPI/SA Mass Ratios	α-Helix (%)	β-Sheet (%)	β-Turn (%)	Random Coil (%)
1:0	24.83 ± 0.06 ^a^	13.87 ± 0.15 ^e^	14.64 ± 0.05 ^c^	44.05 ± 0.15 ^c^
1:0.1	23.67 ± 0.07 ^b^	15.61 ± 1.07 ^d^	15.42 ± 0.23 ^bc^	43.92 ± 1.76 ^bc^
1:0.2	21.03 ± 0.53 ^c^	17.94 ± 0.27 ^c^	17.09 ± 0.81 ^ab^	40.10 ± 0.35 ^b^
1:0.3	20.36 ± 0.71 ^c^	18.93 ± 0.80 ^bc^	18.29 ± 1.76 ^a^	40.97 ± 0.95 ^b^
1:0.4	20.79 ± 1.14 ^c^	19.92 ± 0.34 ^b^	16.66 ± 0.44 ^a^	39.60 ± 0.37 ^a^
1:0.5	17.72 ± 0.21 ^d^	25.02 ± 0.56 ^a^	17.91 ± 0.89 ^a^	38.19 ± 0.53 ^a^

Note: Comparisons were carried out between values of the same column, values with different letters indicate a significant difference at *p* ≤ 0.05.

## Data Availability

The data presented in this study are available in [Exploration of pea protein isolate-sodium alginate complexes as a novel strategy to substitute sugar in plant cream: Synergistic interactions between the two at the interface].
